# Use of minimally invasive autopsy during the COVID-19 pandemic and its possibilities in the context of developing countries

**DOI:** 10.1371/journal.pntd.0009629

**Published:** 2021-08-04

**Authors:** Deborah Nunes Melo, Tania Mara Coelho, Giovanna Rolim Pinheiro Lima, Carolina Gomes Fernandes, Bruno Cavalcante Fales de Brito Alves, Fernanda Montenegro de Carvalho Araújo, Renata Aparecida de Almeida Monteiro, Jaume Ordi, Paulo Hilário do Nascimento Saldiva, Luciano Pamplona de Góes Cavalcanti

**Affiliations:** 1 Programa de Pós-graduação em Patologia, Universidade Federal do Ceará, Fortaleza, Brasil; 2 Serviço de Verificação de Óbitos Dr Rocha Furtado, Fortaleza, Brasil; 3 Programa de Pós-graduação em Saúde Coletiva, Universidade Federal do Ceará, Fortaleza, Brasil; 4 Hospital São Jose de Doenças Infecciosas, Fortaleza, Brasil; 5 Faculdade de Medicina, Centro Universitário Christus, Fortaleza, Brasil; 6 Fundação Oswaldo Cruz, Fortaleza, Brasil; 7 Departamento de Patologia, Faculdade de Medicina da Universidade de São Paulo, São Paulo, Brasil; 8 Department of Pathology, Hospital Clínic, Universitat de Barcelona, Barcelona, Spain; 9 ISGlobal, Barcelona Institute for Global Health, Hospital Clínic, Universitat de Barcelona, Barcelona, Spain; National Institute for Communicable Diseases, Johannesburg, South Africa, SOUTH AFRICA

Coronavirus Disease 2019 (COVID-19) cases have spread quickly across the globe, but the deaths are distributed unevenly. These differences are related to several reasons, including the lack of access to diagnosis and socioeconomic status [1–3].

In this scenario, despite the advances in diagnostic methods in recent decades, a well-performed autopsy remains the gold standard methodology for diagnosing the cause of death [4].

The value of the autopsy has been shown in recent epidemics such as dengue, influenza A/H1N1, Zika, HIV, and yellow fever [5–9]. The autopsy findings helped to clarify not only the basic cause of death, but also the pathophysiology of the disease. These discoveries were possible even after many years when new technologies became available, as exemplified by the studies on paraffin-preserved material from the Spanish flu pandemic of 1918 [10].

Also, autopsies make an indispensable contribution to medical education and training. They provide a unique situation to observe systemic manifestations of different diseases, providing the basis for a medical training.

However, the number of autopsies performed globally is decreasing progressively. This phenomenon is partly due to the lack of limited resources and technical challenges, especially in developing countries. Cultural barriers and the reluctance of families to provide informed consent are additional factors contributing to the decline in autopsy numbers.

Moreover, due to the COVID-19 pandemic, some countries have decided not to allow complete autopsies, which limits the proper investigation of the disease pathophysiology and the death confirmations that were not diagnosed during clinical evolution [11]. In this scenario, some countries are expanding the use of verbal autopsy complemented by specific laboratory tests with good results [12]. However, although verbal autopsy provides a broad approach, its performance for etiological diagnosis is still limited, as it may misclassify some deaths caused by infectious diseases [13].

Thus, the minimally invasive autopsy (MIA) has emerged as an innovative strategy. It is a simple systematic collection technique that utilizes tissue samples from various organs and body fluids, configuring a fast, nondisfiguring procedure, with easy technical applicability that can provide robust data for surveillance, especially in regions with limited resources, being useful to guide strategies for prevention, control, and treatment of diseases [4,14–16].

In addition to health professionals considering it more acceptable, this technique stands out for overcoming the low acceptability and feasibility of a complete autopsy in some regions, which contributes to reducing the stigma of more invasive current practices [4,17].

In the study carried out in Southeastern Brazil during the yellow fever epidemic of 2018, the ultrasound-guided MIA (MIA/US) proved to be effective in both diagnosing the underlying disease and the cause of death and had a 100% diagnostic agreement when compared with conventional autopsy [9]. Similar findings were recently reported in Barcelona with 6 deaths by COVID-19, but without the use of an image resource [16].

The application of imaging techniques may create the impression that MIAs are expensive. However, the adaptation of methodologies and adequate personnel training proved cost-effective diagnostic tools in less developed regions [18,19].

Specifically, in the case of COVID-19 pandemic, in addition to being efficient in establishing the diagnosis of infections, it is a method that offers more safety to the professionals and can be performed with the body closed and surrounded by a plastic cover.

In 2020, the first autopsies using MIA technique were promoted in COVID-19 victims by the project Plataforma de Imagens na Sala de Autópsia (PISA), in the city of São Paulo. A year later, aiming to clarify the role of Severe Acute Respiratory Syndrome Coronavirus 2 (SARS-CoV-2) in lung and systemic injures, MIA was started in the state of Ceará, at the Serviço de Verificação de Óbitos Dr Rocha Furtado [20,21].

The wide acceptance of MIA in combination with simple imaging methods such as ultrasound scan offers a readily available, affordable, and safe postmortem diagnostic technique. Implementing MIA in developing countries can increase the accuracy of epidemiological surveillance indicators and overcome several barriers that prevent the performance of complete autopsies.

This tool can play an important role in improving the surveillance of causes of death in locations where infectious diseases are a common cause of mortality. The application of MIA should not eliminate the need of reinforcing death verification services or performing public verbal autopsies, even though it demonstrates limitations at the individual level, but which are still useful in low-budget situations. It is necessary to increase access to this technique, making it possible to expand the knowledge about the pathophysiology of these emerging diseases that lead thousands of people to death. In addition, its use can expand the range of pathologies that can be seen in biopsies, in the detection of emerging diseases, and even in the diagnosis of chronic diseases.
